# Neutrophil and macrophage zonation in liver disease: from spatiotemporal dynamics to advanced computational analysis

**DOI:** 10.3389/fimmu.2026.1767691

**Published:** 2026-02-24

**Authors:** Hyeree Kim, Nico Lachmann

**Affiliations:** 1 Department of Pediatric Pneumology, Allergology and Neonatology, Hannover Medical School, Hannover, Germany; 2 RESIST, Cluster of Excellence (EXC 2155), Hannover Medical School, Hannover, Germany; 3 Biomedical Research in End Stage and Obstructive Lung Disease Hannover (BREATH), German Center for Lung Research (DZL), Hannover, Germany; 4 Center of Translational Regenerative Medicine, Hannover Medical School, Hannover, Germany; 5 Fraunhofer Institute for Toxicology and Experimental Medicine ITEM, Hannover, Germany

**Keywords:** AI-enhanced analysis, liver zonation, macrophage, multi-omics, neutrophil, single cell transcriptomics, single nuclei transcriptomics, spatial transcriptomics

## Abstract

Liver disease progression is profoundly shaped by the spatial and temporal dynamics of innate immune cells, particularly neutrophils and macrophages. Recent advances in single-cell and spatial omics, intravital imaging, and multiplexed histology have revealed how these cells exhibit distinct zonation patterns along the portal-central axis and undergo dynamic reprogramming in response to injury, infection, and metabolic stress. Neutrophils preferentially accumulate in necrotic or pericentral zones, whereas macrophage subsets adopt diverse zonal identities and display remarkable plasticity, collectively orchestrating inflammation and tissue repair. In this review, we consolidate current knowledge on neutrophil and macrophage zonation in liver disease, emphasizing their roles in shaping pathophysiology and clinical outcomes. We also briefly outline how emerging technologies are refining our understanding of immune microanatomy and may pave the way for precision hepatology.

## 1 Introduction

The liver is not only a central hub of systemic metabolism and xenobiotic detoxification but also a uniquely structured immune organ that continuously interfaces with gut-derived antigens, metabolites, and circulating immune cells (1). Owing to its direct vascular connection with the intestine through the portal vein, the liver is exposed to a constant influx of nutrients, microbial products, and inflammatory signals (2, 3). This anatomical arrangement underlies lobular zonation and the spatial organization of immune cells across the liver lobule.

The hepatic lobule, organized along the portal-central axis, contains finely graded microenvironmental transitions in oxygen tension, metabolic activity (4), nutrient availability, and hormone exposure. These gradients, together with zone-specific stromal and endothelial cues (5), give rise to spatially specialized transcriptional and metabolic programs in both parenchymal and non-parenchymal cells, thereby establishing the foundation for immune zonation (6–11).

The conceptual origins of liver zonation date back to the 1950s and 1960s, when Rappaport introduced the acinus model to explain heterogeneous physiological conditions within the lobule (12, 13). He proposed that portal venous inflow generates progressive gradients in oxygen, substrates, and metabolites as blood flows toward the central vein, leading to the tripartite division of periportal (zone 1), midzonal (zone 2), and pericentral (zone 3) regions. This paradigm first recognized hepatocyte functional heterogeneity despite uniform histological appearance.

Subsequent biochemical and histochemical studies from the 1970s to the 1990s substantiated this framework by demonstrating that key metabolic pathways display discrete spatial compartmentalization (14–17). Gluconeogenesis, ureagenesis, and β-oxidation are enriched in periportal hepatocytes, whereas glycolysis, lipogenesis, and cytochrome P450-mediated xenobiotic metabolism predominate in pericentral cells. These observations established zonation as a fundamental organizing principle of hepatic physiology, underpinning functions ranging from glucose homeostasis to lipid and ammonia metabolism.

The mechanistic basis of zonation was further elucidated in the 2000s. Wnt/β-catenin signaling emerged as a central determinant of pericentral identity (18), with central-vein-associated endothelial and stromal cells producing Wnt ligands (e.g. Wnt2, Wnt9b) (19–22) and potentiators such as R-spondin3 (23, 24). These signals sustain the transcriptional program of zone 3 hepatocytes, whereas lower Wnt activity in periportal regions permits the expression of zone 1-specific metabolic pathways (18). Thus, zonation is now understood as a dynamically regulated transcriptional system maintained by spatially restricted niche signals, rather than a passive outcome of hemodynamic gradients alone.

Recent advances in single-cell (20, 25–28) and spatial transcriptomics (7, 29–32), together with intravital microscopy (3, 33, 34), have expanded this view by demonstrating that zonation extends beyond classical metabolic domains. Immune zonation is tightly shaped by the spatial gradients of antigen exposure generated along the portal-central axis. As blood flows from the portal triads toward the central vein, hepatocytes and non-parenchymal cells adopt distinct spatial states and establish zone-specific patterns of innate immune surveillance (3, 35). Within this spatially graded microenvironment, resident immune sentinels such as Kupffer cells (KCs) and liver sinusoidal endothelial cells (LSECs) balance tolerance to continuous gut-derived antigen stimulation with rapid innate immune activation upon detection of pathogenic bacteria or danger-associated molecular patterns (DAMPs) (2, 36, 37). These zonated differences in antigen exposure, pattern recognition receptor (PRR) expression, phagocytic capacity, and cytokine signaling along the portal-central axis give rise to regionally specialized immune microenvironments within the hepatic lobule (7, 30).

Extending this concept beyond metabolic specialization, the modern understanding of liver zonation is now viewed as an integrated spatial biology framework in which microenvironmental gradients and niche-specific signaling collectively determine the zonated pattern. This spatial organization underlies key aspects of physiological homeostasis and fundamentally shapes the initiation, progression, and resolution of liver diseases. With the emergence of spatial multi-omics technologies, immune zonation is being mapped with high resolution (32), providing new insights into how microanatomical context orchestrates hepatic immune function.

Zonation-based organization of immune cells is essential for hepatic homeostasis, while its perturbation in disease leads to an imbalance in immune cell positioning and function (32). KCs, positioned within the sinusoidal lumen, are particularly sensitive to the metabolic and immunological gradients along the portal-central axis and execute innate immune surveillance in response to blood-borne stimuli (38).

High-resolution spatial systems have revealed that hepatic immune cell organization is more finely partitioned and dynamically regulated than previously appreciated. Neutrophils preferentially accumulate in specific damage zones such as necrotic lesions (3, 31, 39, 40) or periportal inflammatory foci, whereas macrophage subsets acquire region-specific identities and functional programs that differentially contribute to regeneration (7, 41), fibrosis (28, 42), and inflammation (3, 35, 42–47). Furthermore, infection, metabolic stress, and toxic injury induce rapid reprogramming of these immune populations, reshaping communication networks across the lobule. These findings redefine how hepatic innate immunity is spatially coordinated in health and disease.

This emerging spatial immunobiology has important clinical implications. Immune cell rearrangements within cirrhotic nodules, zone-specific regenerative responses in acute liver injury, and inflammatory remodeling in metabolic disease all correlate with disease severity and clinical outcomes (48). These insights indicate that spatial features of immune organization – such as cell location, state, and interaction patterns – may help refine disease stratification and guide the development of more targeted therapeutic approaches.

This review synthesizes current knowledge on the spatial and temporal dynamics of neutrophils and macrophages during liver disease progression, examining how zonated immune regulation contributes to inflammation, tissue injury, fibrosis, regeneration, and host defense. We also highlight how rapidly advancing spatial omics and artificial intelligence (AI)-enhanced analytic frameworks (40, 49–55) are redefining the microanatomy of hepatic immunity, ultimately supporting the development of precision hepatology grounded in disease mechanisms.

## 2 Neutrophil zonation in liver disease

### 2.1 Spatial distribution of neutrophils in homeostasis and disease

Neutrophils are short-lived cells that are delivered to tissues in periodic waves through circadian release from the bone marrow. In mice, CXCR4-CXCL12 signaling mediates bone marrow retention, whereas CXCR2-CXCL1/2 signaling promotes rhythmic egress (56) and resting-phase (ZT2) infiltration into the liver, where neutrophil elastase enhances hepatocyte *Bmal1* and *Clock* expression and lipogenesis. In humans, only the correlation of circadian co-oscillation with *ELANE* and *BMAL1* has been observed rather than causality (57). Consequently, their spatial distribution within the liver is unlikely to reflect stable residence and is plausibly determined by where incoming neutrophils are preferentially captured, retained, or cleared (58, 59).

The hepatic vasculature further reinforces their spatial patterning. Unlike in most organs, neutrophils in the liver bypass selectin-mediated rolling and instead undergo direct adhesion or mechanical trapping within low-shear sinusoids (60). This unique entry mechanism is inherently shaped by a sinusoidal structure, which features a discontinuous endothelial lining that facilitates physiological neutrophil capture. As a result, the hepatic zonated architecture not only influences whether neutrophils enter but also where they localize and how long they persist.

In the steady-state liver, neutrophils are relatively enriched in periportal regions, where they coexist with pathogen-sensing Kupffer cells (2, 36, 37). During liver injury, neutrophil spatial dynamics are reshaped in an insult-dependent manner (58). Sterile toxic injury and partial hepatectomy preferentially drive pericentral accumulation, whereas microbial or bile acid-related signals promote periportal activation (31, 61). Thus, neutrophil zonation in the liver reflects a dynamic balance between wave-like systemic supply and local hepatic cues that regulate recruitment, retention, and egress across physiological and pathological states.

### 2.2 Zonal accumulation in necrotic and pericentral regions

Neutrophil infiltration becomes particularly prominent in liver diseases where injury is spatially restricted. Toxic and metabolic injuries, such as APAP hepatotoxicity (40, 41), carbon tetrachloride (CCl_4_) exposure (41), ischemia-reperfusion (I/R) injury (40), and metabolic dysfunction-associated steatotic liver disease (MASLD/MASH) (39), preferentially damage the pericentral region, creating hypoxic, reactive oxygen species (ROS)-rich, DAMP-dense microenvironments that strongly attract neutrophils. Live imaging using LysM-eGFP mice has demonstrated that neutrophils crawl along sinusoidal endothelium using β2-integrins and follow hierarchical chemotactic cues, first sensing CXCR2 ligands near necrosis and then migrating toward mitochondrial formyl peptides released by dying hepatocytes (3, 62).

As neutrophils reach necrotic pericentral foci, their directed migration transitions to nondirectional patrolling, enabling them to scan the injury bed even when chemokine gradients diminish (63). This selective neutrophil enrichment amplifies monocyte recruitment signals, setting the stage for the accumulation of inflammatory and reparative macrophages that orchestrate tissue remodeling (64, 65).

Neutrophil adhesion mechanisms also exhibit zonal and context-dependent variability. During systemic inflammation, CD44-hyaluronan interactions dominate neutrophil arrest in hepatic sinusoids (66), whereas local chemoattractants shift reliance toward ICAM-1/integrin-mediated adhesion (63, 67). These dynamic adaptations were validated through endotoxemia models (66) highlight that neutrophil accumulation in specific zones is not passive trapping but an actively regulated, stimulus-dependent process.

### 2.3 Functional consequences: tissue injury, inflammation and repair

Zonated neutrophil accumulation has profound consequences for the progression of liver injury. In pericentral necrotic regions, neutrophils amplify tissue damage by releasing ROS, proteases, and pro-inflammatory cytokines, thereby exacerbating hepatocellular damage (58, 68, 69). Their rapid recruitment functions as a spatial checkpoint for subsequent immune escalation, as shown in Con-A-induced hepatitis (70), where early neutrophil activation through L-selectin shedding and ROS production facilitates CD4^+^ T-cell infiltration (60). These observations position neutrophils as zone-specific “gatekeepers” that translate microanatomical context into coordinated inflammatory and adaptive immune responses.

*In vivo* imaging has further revealed that neutrophils produce neutrophil extracellular traps (NETs) in response to endotoxin or bacterial stimuli (71, 72). NETs, DNA-protein lattices with microbicidal properties (59), also exhibit potent pro-inflammatory activity in the liver (73). During I/R injury, DAMPs, such as HMGB1 and IL-33 originating from stressed hepatocytes and LSECs, drive robust neutrophil infiltration and NET formation (40, 49, 68, 74). Superoxide-mediated activation of TLR4-NOX pathways provides an additional trigger (75). Interventions that degrade NETs by Dnase or inhibit their formation by PAD4 inhibitors markedly attenuate liver inflammation (72, 74), underscoring NETosis as a therapeutic target in I/R injury.

Neutrophils also contribute to tissue remodeling and fibrogenesis through zone-dependent mechanisms. In pericentral injury models, ROS and DAMP accumulation strongly induce NETosis. NETs sustain inflammatory signaling and augment monocyte recruitment, and the associated release of histones, proteases, and chemokines activate hepatic stellate cells (HSCs). This environment favors the transition of pericentral HSCs into collagen-producing myofibroblasts, key drivers of progressive fibrosis. Blocking NET formation reduces NASH-HCC progression and mitigates inflammation, implying the pathological relevance of neutrophil-centered zonal networks (73).

Despite their tissue-damaging potential, neutrophils also contribute to repair. Their removal of cellular debris, containment of microbial translocation, and facilitation of macrophage differentiation collectively promote resolution (61). However, the outcome of neutrophil action between injury amplification and repair initiation is largely determined by the zone in which they accumulate, as each lobular region imposes distinct metabolic, stromal, and immunological cues that differentially shape neutrophil function. Pericentral niches enriched in hypoxia, ROS, and hepatocyte-derived DAMPs drive cytotoxic programs and NETosis, whereas periportal environments dominated by microbial signals and tolerogenic cytokines favor debris clearance by neutrophils and restrain excessive neutrophil activation. Thus, the functional imprint of neutrophils is inseparable from the hepatic spatial organization, highlighting zonation as a critical determinant of inflammatory dynamics, fibrogenic progression, and regenerative trajectories.

## 3 Macrophage zonation and plasticity

### 3.1 Distinct zonal identities of resident and recruited macrophages

KCs are the dominant tissue-resident macrophages of the liver and are positioned primarily within periportal to midzonal sinusoids (26, 39). Their asymmetric distribution is not developmentally pre-determined but arises from sustained exposure to gut-derived microbial products via MyD88-dependent signaling in LSECs (3), along with chemokine gradients that retain KCs in zone 1 (39). Periportal MARCO^+^ F4/80^+^ CLECF4^+^ KCs specialize in clearing bacteria (3, 76) entering through the portal vein and maintaining immune tolerance by producing IL-10 and scavenging microbial metabolites (3). Under pathological conditions, however, KC identity and localization are altered. During steatosis, VSIG4^+^ FOLR2^+^ CD163^+^ CD169^+^ KCs are enriched in zone 2 (39), whereas acute liver injury is characterized by the emergence of MARCO^-^ F4/80^+^ KCs in zone 3 (3). In chronic injury, KCs enriched in zone 3 progressively acquire a LAM-like phenotype, characterized by expression of TREM2^+^ GPNMB^+^ TIM4^+^ CLEC4F^+^ F4/80^int^ CD36^hi^ (41). Although early single-cell studies debated KC heterogeneity (8, 26), recent spatial profiling has clarified that KCs possess zonally distinct identities shaped by their microanatomical context (39, 77).

In homeostasis, KCs remain largely immobile and maintain liver immune equilibrium by removing pathogens, apoptotic cells, and toxins, while suppressing unnecessary inflammation (39, 78). However, their distribution is prone to modulation by physiological stress and disease. Age-related functional decline (79), chronic inflammation, and metabolic injury result in marked KC depletion, particularly in periportal niches, which subsequently replenished by circulating monocytes (80). This process is dynamically regulated rather than stochastic, as monocytes trafficking and tissue infiltration follow circadian rhythms coordinated by cell-intrinsic clocks and hepatic cues (81, 82). The zonated hepatic microenvironment determines whether these incoming monocytes acquire tolerogenic KC-like identity or retain inflammatory programs, particularly in the milieu dominated by pathogen-associated molecular pattern (PAMP) or DAMP signals, pro-inflammatory cytokines, and metabolic stress. These dynamics underscore macrophage identity as a spatially governed state.

### 3.2 Dynamic reprogramming in response to injury and metabolic stress

Acute and chronic liver injury deplete resident KCs, allowing monocytes to migrate toward the vacant KC niches, where they differentiate into monocyte-derived macrophages (MoMFs) (46). In fibrosis-prone regions, particularly pericentral zone 3, these MoMFs accumulate in proximity to HSCs (42), where they contribute to the early phases of fibrogenesis and inflammation. Spatial omics data support this pattern, revealing macrophage-HSC niches forming specifically around injury-associated fibrotic bands (42).

Metabolic and immune perturbations further diversify the reprogramming trajectories of MoMFs (35, 44). In MASLD/MASH (3, 41, 43, 45, 46) and cholestatic disorders [e.g. primary biliary cirrhosis (83, 84) and primary sclerosing cholangitis (83)], MoMFs accumulate in distinct zones depending on the dominant insult. Some MoMFs localize periportally, contributing to ductular reaction (83), while others cluster near steatotic or pericentral regions in response to lipid overload or hepatocyte stress (39). These findings highlight that macrophage zonation is not static but reflects the dynamic mapping of macrophage subsets to metabolic, immunological, and stromal cues present in specific hepatic regions.

Furthermore, depending on gradients of oxygen, metabolites, cytokines, and DAMPs, MoMFs may adopt inflammatory phenotypes, lipid-associated macrophage (LAM) states, or transition toward KC-like identity (41, 43–46). Cholangiocyte-derived signals can recruit MoMFs to periportal areas during ductular reaction after 3,5-diethoxycarbonyl-1,4-dihydrocollidine (DDC) injury (85, 86), whereas steatotic hepatocytes drive MoMF accumulation in pericentral or panzonal patterns in MASLD/MASH (41, 43, 45, 46). Thus, macrophage plasticity reflects not only their ontogeny but also the zonated biochemical landscapes into which they are recruited.

### 3.3 Zonal macrophage functions in fibrosis, regeneration, and infection

As fibrosis progresses, MoMFs diversify into specialized subsets, including LAMs and scar-associated macrophages (SAMs), characterized by TREM2, CD9, and osteopontin (28, 41, 43, 46, 47). These populations preferentially localize to collagen-rich, inflamed, or ECM-remodeling regions (28). Spatial transcriptomics further demonstrated that TREM2^+^ macrophages form structured fibrotic niches (8, 41, 46, 84, 87), and circulating soluble TREM2 correlates with MASH severity (87), highlighting their combined fibrogenic and metabolic roles.

In advanced fibrosis and cirrhosis, resident TIMD4^+^/MARCO^+^ KCs progressively diminish (32), whereas SAMs expressing TREM2 and CE9 accumulate along pericentral and bridging fibrotic septa (28). These SAMs directly interact with HSCs through TGF-β, PDGF, and CCL/MMP signaling, promoting myofibroblast activation and extracellular matrix deposition (88). Their spatial co-localization with HSCs within pericentral fibrotic niches provides a mechanistic link between zonated macrophage recruitment, HSC pathogenic transformation, and progressive collagen production.

Macrophage plasticity also influences regeneration. Following KC depletion, MoMFs repopulate hepatocellular interfaces and support repair through phagocytosis (45), efferocytosis (41), and secretion of pro-regenerative mediators (89). In fibrotic liver, MoMFs can fuse to form multinucleated macrophage syncytia, a process that restores KC-like functions by enhancing phagocytic capacity. This fusion-mediated reprogramming is critical for regeneration, as failure to form syncytia leads to impaired debris clearance, persistent inflammation, and defective restoration of macrophage zonation (34).

Within the fibrotic niche microenvironment, the relocation of HSCs around remodeled and enlarged vessels appears to provide instructive cues that facilitate the acquisition of KC-like features by syncytia, enabling them to capture more particles than individual KCs in their native sinusoidal setting. These findings suggest that KC-like identity can be rebuilt *de novo* from recruited monocytes under appropriate spatial cues (34).

Zonation also governs macrophage responses to infection. Periportal MARCO^+^ KCs restrict bacterial dissemination by capturing gut-derived microbes (39, 76), whereas pericentral macrophages become more activated in viral infections or in metabolic stress conditions (35, 44, 83). In both contexts, macrophages act as spatially anchored regulators linking local hepatocyte damage, neutrophil recruitment, and adaptive immune activation.

Altogether, these zonal macrophage circuits underscore the liver’s microanatomy as a spatially compartmentalized immune organ in which macrophage identity, function, and plasticity are continuously redefined by the architecture and metabolic gradients of the lobule.

## 4 Discussion

Building on emerging evidence of intrahepatic neutrophils and macrophages heterogeneity (**Tables 1**, **2**), recent computational advances have profoundly enhanced the spatial and functional resolution at which immune zonation can be interrogated (32). Advanced computational analysis applied to intravital microscopy and high-dimensional histology (90) now enables automated tracking of neutrophil dynamics within the sinusoidal network, allowing precise investigation of how neutrophils interact with microvasculature under homeostatic, necrotic, and inflammatory conditions (91). These approaches reveal subtle zone-specific differences in crawling velocity, adhesion patterns, and sinusoidal trapping-features that were previously not feasible to measure manually. Physics-informed deep learning applied to light-field intravital microscopy has further enabled long-term, high-resolution imaging of Ly6G^+^ neutrophils and F4/80^+^ KCs within intact vascular niches, revealing their interactions in I/R and toxic injury models (40, 55, 92).

**Table 1:** Human intrahepatic neutrophil and macrophage heterogeneity across physiological and pathological conditions.

Species	Conditions	Key features of intrahepatic neutrophils and macrophages	Technology	Year	References
Human	Homeostasis	CD68^+^ MARCO^+^ tolerogenic, non-inflammatory KC concentrated in PP	scRNA-seq, snRNA-seq (total 6,000 cells)	2018	(26)
Human	Homeostasis	Non-inflammatory MΦ expressing *CD86*, *LYZ*, *MARCO*, *CD163* in PV Inflammatory MΦ expressing *CD86*, *LYZ*, *MARCO*, *CD163* in CV	scRNA-seq (29,432 cells), snRNA-seq (43,863 cells), Visium	2022	(29)
Human	Homeostasis	VSIG4^+^ FOLR2^+^ CD163^+^ CD169^+^ KC expressing *CD5L* in mid zone CD68^+^ VSIG4^-^ MΦ in the liver capsule, in close proximity to CV, PV, BDs	Visium, molecular cartography, scCITE-seq, snRNA-seq (8,000-10,000 cells), MICS	2022	(39)
Human	Homeostasis	MARCO^-^ CD5L^-^ CD68^lo^ non-inflammatory MΦ expressing *CD74* in PP and also dispersed through the lobules KC expressing *CD74*, *CD5L*, *MARCO* in scattered more diffusely through the lobules Enriched MARCO^+^ CD5L^+^ CD68^+^ VSIG4^+^ KC in PP	MERFISH, snRNA-seq (~310,000 cells)	2025	(32)
Human	Fibrosis	Spatially recruited TREM2^+^ CD9^+^ MNDA^+^ SAMs in collagen-positive scar regions	scRNA-seq, smFISH (total 100,000 cells)	2019	(28)
Human	Fibrosis	Enriched CD9^+^ TREM2^+^ SPP1^+^ GPNMB^+^ FABP5^+^ CD63^+^ SAMs at scarring edges, clustered with *MMP9*-expressing neutrophils	scRNA-seq, CycIF	2023	(47)
Human	NAFLD/NASH AH PSC PBC	IBA1^+^ CD16^lo^ CD163^lo^ MΦ in the vicinity of BD	InSituPlex Ultivue	2023	(83)
Human	PSC	Enriched TREM2^+^ monocyte-like MΦ within fibrotic niche MHCII^+^ LAM-like MΦ/KC in PC Activated MΦ in PP VCAM1^+^ KC-like cells in both PC and PP (but only increased in PP) Recruited SAM-like MoMF in the center of fibrotic regions, whereas KC were localized outside of the scar regions	scRNA-seq (107,542 cells in NDD; 47,156 cells in PSC; 18,240 cells in PBC), snRNA-seq (23,000 cells in PSC; 20,202 cells in PBC), Visium, Nanostring GeoMx DSP	2024	(84)

AH, alcoholic hepatitis; BD, bile duct; CV, central vein; KC, Kupffer cell; MΦ, macrophage; MICS, MACSima Imaging Cyclic Staining; MERFISH, multiplexed error robust fluorescent in situ hybridization; MoMF, monocyte-derived macrophage; NAFLD, nonalcoholic fatty liver disease; NASH, nonalcoholic steatohepatitis; NDD, neurologically deceased healthy donor; PBC, primary biliary cholangitis; PP, periportal; PSC, primary sclerosing cholangitis; PV, portal vein; SAM, scar-associated macrophage; scCITE-seq, single-cell cellular indexing of transcriptomes and epitopes by sequencing; scRNA-seq, single-cell RNA sequencing; smFISH, single molecule fluorescence in situ hybridization; snRNA-seq, single nucleus RNA sequencing.

**Table 2:** Mouse intrahepatic neutrophil and macrophage heterogeneity across physiological and pathological conditions.

Species	Conditions	Key features of intrahepatic neutrophils and macrophages	Technology	Year	References
Mouse	Homeostasis	KC expressing Clec4f in PP	scRNA-seq	2021	(90)
Mouse	Homeostasis	KC preferentially localized in PP and mid zones, without a strong zonation pattern	scRNA-seq (16,900 cells), scATAC-seq (9,702 cells), scATAC-seq + scRNA-seq (12,898 cells), MICS, smFISH, AI	2024	(52)
Mouse	Homeostasis	LAMs expressing Gpnmb, Spp1 in PP, further defined by more Il1b expression compared with KC MΦ expressing Cd207 in CV MΦ expressing Ccr2, Chil3 at the PV and CV, resembling transitioning Mo Enriched MΦ expressing Gpnmb around the bile ducts (termed as bile-duct LAMs) KC in PP, whereas sporadically distributed Mo	Visium, molecular cartography, scCITE-seq, snRNA-seq (8,000-10,000 cells), MICS	2022	(39)
	MASLD/MASH	MΦ/Mo in mid-CV LAMs primarily in PC, defined by lower gene expression of Il1b, Tnf, Il10 in WD			
Mouse	Fibrosis	Enriched CD9^+^ TREM2^+^ SPP1^+^ GPNMB^+^ FABP5^+^ CD63^+^ SAMs, at scarring edges, clustered with MMP9-expressing neutrophils Co-localization of Iba1^+^ MΦ and increased S100A8/A9^+^ neutrophils in scarring region	scRNA-seq, CycIF	2023	(47)
Mouse	Fibrosis	F4/80^+^ CLEC4F^+^, CRIg^+^, TIM-4^+^ KC in PP Recruited CX3CR1^+^ MoMF in CV, forming syncytia via CD36	IVM	2023	(34)
Mouse	Fibrosis Liver injury MASH	TREM2^+^ GPNMB^+^ TIM4^+^ CLEC4F^+^ F4/80^int^ CD36^hi^ LAM-like resident KCs in CV, further defined by Mmp12 expression F4/80^+^ TREM2^+^ recruited MΦ in CV	IVM, scRNA-seq, snRNA-seq, Visium	2025	(41)
Mouse	MAFLD	CLEC4F^+^ TIM4^+^ ResKCs, CLEC4F^+^ TIM4^-^ moKCs, and sporadically distributed CLEC4F^-^ MΦ CLEC4F^-^ MΦ in close proximity to the large vessels (CV/PV) in fibrotic zones	scRNA-seq (56,407 cells), metabolomics, lipidomics	2020	(43)
Mouse	Homeostasis	Evenly distributed KC	scRNA-seq (82,168 cells)	2021	(10)
	NAFLD	Accumulated KC around the injured regions to form unique CLS			
Mouse	NASH	SAM expressing TREM2 and CD9 in close proximity to fibrotic region Enriched Ly6C^hi^ CD301b^-^ RM in both PV and CV Enriched Ly6C^lo^ CD301b^+^ RM in closer proximity to CV	scRNA-seq, snATAC-seq (50,000 cells)	2020	(44)
Mouse	Liver injury	Neutrophils preferentially accumulated at damage sites in CV Marco^+^ F4/80^+^ expressing Clec4f KC in PV, defined by gene expression of Il10, Il1rn, Tgfb1 Marco^-^ F4/80^+^ KC in CV	Visium, IVM	2024	(3)
Mouse	Liver regeneration	Infiltrated neutrophils (expressing S100a8, S100a9, Cxcr2, F13a1, Fgr) expanding from the midzonal to PC area upon PHx Enriched Bmp10-Acvrl1 interaction in KC in the midzonal area	Stereo-seq, scRNA-seq (473,290 cells), smFISH, multiplex RNAscope	2024	(31)
Mouse	Obesity-related steatohepatitis	Prominent F4/80^+^ MΦ infiltration in PP	scRNA-seq (~2,500 cells/sample)	2020	(35)

AI, artificial intelligence; CLS, crown-like structure; CV, central vein; CycIF, cyclic immunofluorescence; IVM, intravital microscopy; KC, Kupffer cell; LAM, lipid-associated macrophage; MΦ, macrophage; MAFLD, metabolic dysfunction-associated fatty liver disease; MASH, metabolic dysfunction-associated steatohepatitis; MICS, MACSima Imaging Cyclic Staining; Mo, monocyte; MoMF, monocyte-derived macrophage; NAFLD, nonalcoholic fatty liver disease; NASH, nonalcoholic steatohepatitis; PHx, partial hepatectomy; PP, periportal; PV, portal vein; SAM, scar-associated macrophage; scCITE-seq, single-cell cellular indexing of transcriptomes and epitopes by sequencing; scRNA-seq, single-cell RNA sequencing; scATAC-seq, single-cell assay for transposase-accessible chromatin using sequencing; smFISH, single molecule fluorescence in situ hybridization; snRNA-seq, single nucleus RNA sequencing; Stereo-seq, spatiotemporal enhanced resolution omics-sequencing; WD, westerndiet.

Deep learning methods have also transformed the characterization of KC zonation states (52). By integrating marker expression, morphological signatures, and sinusoidal topology (50), convolutional neural networks can distinguish periportal tolerogenic KCs, midzonal transitional states, and pericentral stress-responsive populations with high fidelity (53). When coupled with cell-cell proximity analysis, these models reconstruct the spatial rules governing KC interactions with hepatocytes, LSECs, and infiltrating MoMFs (32). This analytical framework has revealed that specific KC states preferentially associate with discrete vascular niches, creating spatially distinct immunoregulatory microenvironments. Deep-learning-assisted three-dimensional (3D) reconstruction frameworks extend these capabilities, enabling the mapping of KCs together with hepatocytes, stellate cells, and the sinusoidal network at single-cell resolution (53).

Beyond image-based mapping, the integration of spatial transcriptomics with AI-enhanced inference tools is redefining how immune communication networks are conceptualized (54, 90). Deep learning models trained on multi-omic datasets can infer ligand-receptor interactions within zonated niches, disentangling how cytokine, chemokine, and growth factor signaling differ across the portal-central axis. A recent study employing enhancer-resolved spatial multi-omics demonstrated that deep neural networks can reconstruct zonated gene-regulatory networks, revealing enhancer-driven programs that shape immune cell identity more precisely than bulk or single-cell data alone (52).

Collectively, these technological advances illustrate how advanced spatial biology is fundamentally redefining immune zonation. Rather than static maps of cell localization, the liver is now understood as a dynamically encoded spatial immune system, where neutrophils, KCs, and MoMFs continuously respond to microenvironmental cues and engage in structured circuit-level interactions. This integration of advanced computational tools with spatial multi-omics provides the conceptual and analytical foundation for the emerging era of precision hepatology.
